# Insight for pediatricians: Comparing economic trends for firearm related deaths and deaths from motor vehicle crashes

**DOI:** 10.3389/fped.2023.1165301

**Published:** 2023-03-31

**Authors:** Shabih Manzar, Katherine Hoops, Dayanand Bagdure

**Affiliations:** ^1^ Department of Pediatrics, Louisiana State University Health Science Center, Shreveport, LA, United States; ^2^ Department of Anesthesiology and Critical Care Medicine, Johns Hopkins University, Baltimore, MD, United States

**Keywords:** firearm, pediatrics, etiology, motor vechicle crashes, epidemiology

With every school shooting, debate on firearms garners national attention. Pediatricians will have to play an important role in the practice of promoting and integrating positive changes in the environment for children. Pediatricians played an important role in the early years of motor vehicular crashes to draw national attention to the epidemic and impact on children. We provide the perspective of the financial burden due to firearm injuries, contrasting it with vehicular crashes, an additional insight for pediatricians to promote firearm safety.

Since 2013, fatal firearm injuries in children and adolescents have been increasing in United States (1). Lee et al. described a concerning trend for deaths in children, adolescents, and youth ages 1–24 years in the United States (2). In 2017, deaths due to firearm related injuries became the most common cause of death in this age group, surpassing deaths due to motor vehicle crashes. Lee and colleagues analyzed CDC's data from 2000 to 2020 and saw the number of firearm related deaths among children, adolescents and young adults increased from 7.3 per 100,000 persons to 10.28 per 100,000 persons. There is political will and a public cry to address firearm injuries as a public health problem, and research on trends, disparities, risk factors will help guide preventive efforts.

In the United States, since 2016, the economic impact of firearm related deaths, both in terms of medical costs and the statistical value of life lost to death, is consistently greater than 1 billion dollars each year. Millions of dollars are spent caring for youth in emergency departments (3), and billions of dollars are spent on inpatient care (4, 5).

CDC has ensured publicly available data through the Web-based Injury Statistics Query and Reporting System (WISQARS) (6). It is an interactive data visualization and can provide insights in trends in deaths due to various causes of injuries. WISQARS is proving to be an essential tool in identifying nature and extent of injury, fatal and non-fatal burden of such injuries, and prioritization of prevention strategies (7).

To understand the economic impact of firearm injuries from 2015 to 2020, we compared the percentage change to 2015 data for fatalities and total cost (medical and value of statistical life) for both firearm injuries and motor vehicle crashes. After obtaining the data from WISQARS, data for each type of injury for each year was tabulated and the percentage increase to the previous year was calculated. The fatality data is obtained from National Vital Statistics System (NVSS) using the death certificates. For the estimate of cost of injury data is obtained from Pacific Institute of Research and Evaluation.

We found that the percentage change in cost for firearm injuries or motor vehicle crashes corresponded to the mortality in that particular year, higher the mortality that year, higher the cost. Beginning in 2017, we found a striking trend with an increase in fatalities and total cost for firearm injuries and decrease in fatalities and total cost for motor vehicle crashes (Figure 1). It has been shown that since 2017, deaths due to firearm injuries became the leading cause of deaths for children, adolescents, and young adults. In 2020, deaths and total costs for firearm related injuries were 39.2% and 41% respectively, higher than in 2015. In 2019, we observed the largest drop in motor vehicle deaths (12.35%) and total costs (12%). These cost are only for fatal injuries either due to firearms or motor vehicular crashes.

**Figure 1:**
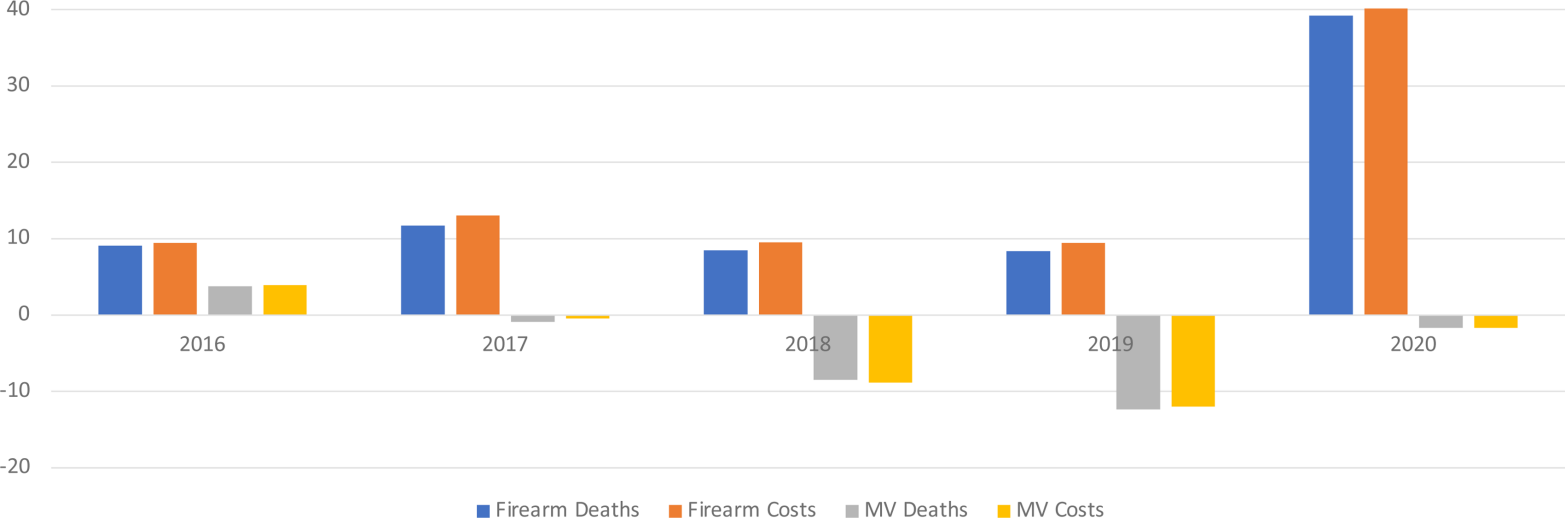
Percentage change in deaths and costs for firearm and motor vehicle fatalities in 1–24 years of age (compared to 2015).

During the COVID-19 pandemic there was a surge in firearm sales and in gun deaths. We have also seen an increase in the use of firearms to commit suicide. Rural children are at increasing risk for using firearms to commit suicide. Urban children are more prone to unintentional or homicidal victims of firearm injuries.

Lee and colleagues (2) present us with a comprehensive public health approach to injury reduction that has been extremely successful in preventing injury-related deaths. This should serve as a model for the public health approach to reduce firearm-related injuries and deaths. The measures implemented to prevent motor vehicle related deaths in the last 20 or more years and their efficacy in achieving that goal sit in stark contrast to the comparatively limited interventions to prevent youth firearm death and injury. Similar to the approach to motor vehicular collisions, the approach to firearm injuries can be divided into three phases to decrease the morbidity and mortality: (1) Pre-event phase (access to mental health services, child access prevention laws, licensing and permit-to-purchase requirements, extreme risk protective orders), (2) Event phase (school safety policies, “gun free zones,” magazine capacity limits, bump stock prohibitions), and (3) post event phase (medical and rehabilitation services, trauma center accreditation, EMS systems). Onus should not fall on a particular group of individuals, but each group will have to play their role if we are to decrease the mortality burden due to firearm injuries. We are seeing an increase in research funding for firearm injuries which will lead to identifying and prioritizing prevention strategies. Policy makers will have evidenced based scientific data to make informed decision for allocation of resources. One of the limitations of the study is the age group (1–24 years) arguing the higher incidence of firearm injuries in young adults and not necessarily in children. Most pediatricians are involved in the care of children and young adults up to 18 or 21 years of age. We used this age group as CDC does provide data in 5-year or 10-year age groups and data was similar to previously published study (2).

While, as pediatricians, we believe that children's lives tragically lost to violence are compelling to drive policy and research, our report also helps to highlight the economic burden of firearm-related deaths and injuries and may further strengthen the case for investment in research and in evidence-based policy initiatives to prevent firearm injuries. Pediatricians will have to harness each opportunity available or each piece of emerging data available to convince policy makers for positive changes as we amplify the voices of our patients, families, and communities that we serve.

## References

[1] Cunningham RM Ranney ML Goldstick JE Kamat SV Roche JS Carter PM. Federal funding for research on the leading causes of death among children and adolescents. Health Aff (Millwood). (2019) 38(10):1653–61. 10.1377/hlthaff.2019.00476 31589521 PMC7039655

[2] Lee LK Douglas K Hemenway D. Crossing lines—a change in the leading cause of death among U.S. Children. N Engl J Med. (2022) 386(16):1485–7. 10.1056/NEJMp2200169 35426978

[3] Gani F Canner JK. Trends in the incidence of and charges associated with firearm-related injuries among pediatric patients, 2006–2014. JAMA Pediatr. (2018) 172(12):1195–6. 10.1001/jamapediatrics.2018.3091 30383089 PMC6583683

[4] Lee J Quraishi SA Bhatnagar S Zafonte RD Masiakos PT. The economic cost of firearm-related injuries in the United States from 2006 to 2010. Surgery. (2014) 155(5):894–8. 10.1016/j.surg.2014.02.011 24684950

[5] Taylor JS Madhavan S Han RW Chandler JM Tenakoon L Chao S. Financial burden of pediatric firearm-related injury admissions in the United States. PLoS One. (2021) 16(6):e0252821. 10.1371/journal.pone.0252821 34161341 PMC8221502

[6] WISQARS (web-based injury statistics query and reporting system). Centers for Disease Control and Prevention. Available at: https://www.cdc.gov/injury/wisqars/index.html (Published December 2, 2021, Accessed July 5, 2022).

[7] Ballesteros MF Webb K McClure RJ. A review of CDC's web-based injury statistics query and reporting system (WISQARS™): planning for the future of injury surveillance. J Safety Res. (2017) 61:211–5. 10.1016/j.jsr.2017.01.001 28454867 PMC5605760

